# Associations between Purine Metabolites and Clinical Symptoms in Schizophrenia

**DOI:** 10.1371/journal.pone.0042165

**Published:** 2012-08-14

**Authors:** Jeffrey K. Yao, Ruth Condray, George G. Dougherty, Matcheri S. Keshavan, Debra M. Montrose, Wayne R. Matson, Joseph McEvoy, Rima Kaddurah-Daouk, Ravinder D. Reddy

**Affiliations:** 1 Veterans Affairs Pittsburgh Healthcare System, Pittsburgh, Pennsylvania, United States of America; 2 Department of Psychiatry, Western Psychiatric Institute & Clinic, University of Pittsburgh Medical Center, Pittsburgh, Pennsylvania, United States of America; 3 Department of Pharmaceutical Sciences, University of Pittsburgh School of Pharmacy, Pittsburgh, Pennsylvania, United States of America; 4 Beth Israel Deaconess Medical Center and Harvard University, Boston, Massachusetts, United States of America; 5 Bedford Veterans Affairs Medical Center, Bedford, Massachusetts, United States of America; 6 Duke University Medical Center, Durham, North Carolina, United States of America; Chiba University Center for Forensic Mental Health, Japan

## Abstract

**Background:**

The antioxidant defense system, which is known to be dysregulated in schizophrenia, is closely linked to the dynamics of purine pathway. Thus, alterations in the homeostatic balance in the purine pathway may be involved in the pathophysiology of schizophrenia.

**Methodology/Principal Findings:**

Breakdown products in purine pathway were measured using high-pressure liquid chromatography coupled with a coulometric multi-electrode array system for 25 first-episode neuroleptic-naïve patients with schizophrenia at baseline and at 4-weeks following initiation of treatment with antipsychotic medication. Associations between these metabolites and clinical and neurological symptoms were examined at both time points. The ratio of uric acid and guanine measured at baseline predicted clinical improvement following four weeks of treatment with antipsychotic medication. Baseline levels of purine metabolites also predicted clinical and neurological symtpoms recorded at baseline; level of guanosine was associated with degree of clinical thought disturbance, and the ratio of xanthosine to guanosine at baseline predicted degree of impairment in the repetition and sequencing of actions.

**Conclusions/Significance:**

Findings suggest an association between optimal levels of purine byproducts and dynamics in clinical symptoms and adjustment, as well as in the integrity of sensory and motor processing. Taken together, alterations in purine catabolism may have clinical relevance in schizophrenia pathology.

## Introduction

There is abundant evidence for impaired antioxidant defense system (AODS) and presence of oxidative stress in patients with schizophrenia (SZ) [1]–[6], suggesting that free radical-mediated pathology may have a role in the pathophysiology of SZ.

Free radicals are reactive chemical species generated during normal metabolic processes involving oxygen and nitric oxide, which in excess can lead to membrane damage. Under physiological conditions the potential for free radical-mediated damage is kept in check by the AODS, comprising a series of enzymatic and non-enzymatic components. These enzymes act cooperatively at different sites in the free radical pathways. We have previously observed that a dynamic state is kept in check during the redox coupling under normal conditions [7]–[8]. By contrast, lack of such correlations in brains of SZ patients point to a disturbance of redox coupling mechanisms in the AODS, possibly resulting from a decreased level of glutathione (GSH) as well as age-related decreases of oxidized GSH and glutathione reductase activities.

In addition to GSH redox coupling mechanism, purine catabolism may be an underappreciated component of the homeostatic response of mitochondria to oxidant stress and may play a critical role in slowing progressive mitochondrial dysfunction in certain disease states [9]. In response to oxidative stress, decreased energy charge or nucleic acid damage, purine metabolism shifts to favor breakdown to xanthine and uric acid. Recently, we [8] have further shown that a shift favorable to Xanthosine production from Xanthine, resulting in decreased uric acid (UA) levels in the first-episode neuroleptic-naïve patients with schizophrenia (FENNS). Specifically, the reduced UA/guanosine ratio was nearly normalized after 4-week of antipsychotic treatment. In addition, there are tightly correlated precursor and product relationships within purine pathways; although some of these correlations persist across disease or medication status, others appear to be lost among FENNS. Taken together, these results suggest that the potential for steady formation of antioxidant UA from purine catabolism is altered early in the course of illness.

In this study, we extend our previous findings [8] to test whether homeostatic imbalance in purine catabolism is associated with specific clinical characteristics observed in FENNS at baseline and/or 4 weeks after antipsychotic treatment.

## Materials and Methods

### First-Episode Neuroleptic-Naïve (FENN) Patients

Twenty five patients were recruited (Table 1) in their first episode of psychosis after they provisionally met DSM-IV criteria for schizophrenia, schizophreniform or schizoaffective disorder based on Structured Clinical Interview for DSM Disorders (SCID). The initial diagnostic assessments were performed at consensus diagnostic conferences including SCID and all clinical data, and attended by research faculty and staff, chaired by one of the authors (MSK or DM). All subjects signed informed consent after a full explanation of the study. The study was approved by both VA Pittsburgh Healthcare System and the University of Pittsburgh Institutional Review Board.

**Table 1:** Subject characteristics of first-episode neuroleptic-naïve patients with schizophrenia.

*Demographical features*	*Male*	*Female*
Number	19	6
Age (yrs, mean ± SD)	21.4±5.5	26.3±10.6
Educations (yrs)	11.8±2.9	12.3±4.6
Weight (lbs)	156.1±43.5	120.0±19.2
Height (inches)	68.2±4.1	64.3±1.7
Body mass index (BMI)	23.9±4.8	20.7±3.0

Blood samples were obtained in patients at baseline (FENNS-BL) prior to the initiation of antipsychotic agents. A second set of blood samples was obtained in the same patient individuals about 4 weeks after initiation of treatment (FENNS-4w) with one or more of the following antipsychotic drugs: risperidone (n = 17), olanzapine (n = 5), quetiapine (n = 2), aripiprazole (n = 1) and haloperidol (n = 2). The number adds up to more than 25 because of polypharmacy.

Clinical symptoms were rated by experienced research clinicians at both time points using standard rating scales. Our primary question concerned the association between purine catabolism and the severity of psychiatric disturbance in patients during the early phase of their illness. Severity of clinical presentation was evaluated using the Global Assessment Scale (GAS), which has a high degree of sensitivity to clinical change over time [10]. The relationships among purine metabolites and specific clinical symptom dimensions were additionally explored and included ratings of positive and negative symptoms (Positive Symptoms scale from the Brief Psychiatric Rating Scale (BPRS) [11]; Scale for Assessment of Negative Symptoms (SANS) [12]) and neurological symptoms (Neurological Evaluation Scale (NES) [13]). As expected, patients showed improvement in clinical symptoms and clinical functioning after 4 weeks of treatment with antipsychotic drugs (Table 2).

**Table 2:** Clinical assessments of first-episode neuroleptic-naïve patients with schizophrenia (n = 25).

Rating scales	Baseline	4-wk follow-up	*p*
Brief Psychiatric Rating Scale (total)*	52.58±8.90	42.92±7.50	<0.001†
Brief Psychiatric Rating Scale–Positive Symptoms	15.72±3.25	11.80±4.29	0.001‡
Scale for Assessment of Negative Symptoms	45.08±10.30	43.00±9.09	0.410†
Global Assessment Scale	30.40±9.10	37.76±11.79	0.001‡

* missing data for 1 patient.

† Paired samples *t*-test.

‡ Related-samples Wilcoxon Signed Rank Test.

### Sample Preparation

All blood samples were collected in the morning after overnight fasting. Samples were prepared for analysis by extraction in acidified acetonitrile and analyzed by liquid chromatography with electrode Coul Array (LCECA) system as previously described [8], [14]–[15]. Briefly, freshly drawn blood with anticoagulant citrate dextrose (ACD) was centrifuged at 750×g for 7 minutes to remove RBC and stored at −80°C freezer. 250 µl aliquot of stored sample was mixed with 1 ml of acetonitrile/0.4% acetic acid at −25°C and vortexed for 45–60 sec, then temperature was brought to −15°C in a cold block, and vortexed again for 30–45 sec. Samples were centrifuged for 15 min at 12,000×g at 4°C. In total, 1 ml of the resulting supernate was transferred to a 2 ml screw top vial and evaporated under vacuum. It is critical that the vacuum is sufficient to freeze the sample during this step. The sample was reconstituted in 200 µl of mobile phase A and 100 µl were loaded onto two autosampler vials, one of which was archived at −80°C. Profiles are stable in acetonitrile extract, dried extract and mobile phase diluted extract.

During the sample preparation, pools were created from equal volumes of aliquots of all samples. All assays were run in sequences that include 10 samples, authentic reference standard mixtures of 80 known compounds, pools of all samples and duplicate preparations of the same sample. Duplicates are spaced at short and long intervals through the run to reflect the performance of the total data base. Run orders of all samples in this study were randomized. The sequences minimized possible analytical artifacts during further data processing. Pools and duplicates were used to access the precision of the entire data set. In addition, the pools were used as references for time normalization (stretching). A practical advantage of LCECA for this study is the relative freedom from maintenance events. This is important for the generation of consistent databases from large numbers of samples over extended time periods. In our earlier work we have run LCECA continuously for 24 h per day over 6 months.

### High-Pressure Liquid Chromatography Coupled With Electrochemical Coulometric Array (LCECA) Detection

The LCECA method used in this work has been described earlier [8], [14]–[16]. Briefly the liquid chromatographic method employs an A mobile phase (10.3 g l^−1^ sodium pentane sulfonate, 5 ml l^−1^ glacial acetic acid) and a B mobile phase (methanol/acetonitrile/isopropanol 8/1/1, 8 g l^−1^ lithium acetate, 20 ml l^−1^ glacial acetic acid). A gradient is run from 100% A to 100% B over 120 min. The electrochemical array of 16 series coulometric detectors is set from 0 to 900 mv in equal 60 mv increments from detector 1–16. In this mode a compound passing through a coulometric electrode is oxidized by 100% of the thermodynamically possible amount. This results in a characteristic signature for a compound expressed as a ratio on sequential electrodes. This ratio provides a high degree of qualitative certainty, which can be set for any particular study [17]. The gradient and detector conditions typically provide responses at the 500 pg ml^−1^ level (5 pg on column) for ca 1500–2000 compounds in biological samples. In comparison with Mass Spectrometry (MS) a specific thermodynamically determined response ratio and retention time in an LCECA method does not carry as much qualitative certainty as an accurate mass peak or fragmentation pattern in MS/MS. However, in comparison with MS for the classes of compounds measurable on LCECA the sensitivity of ca 500 pg ml^−1^ is typically one to two orders of magnitude lower than can be achieved with MS. As an example we conducted a study directed at identifying metabolites implied by the presence of multiple responses in an LCECA method following Huntington's disease patients treated with phenyl butyrate [18]. The LCECA method employed 40 µl of plasma. It was necessary to concentrate and fractionate 4 ml of plasma to obtain sufficient material for qualitative identification in a parallel LCECA/LCMS system. The LCECA method with 100% efficient electron transfer also has an inherent quantitative control based on integration of the total coulombs of the peaks [18] and calculation of quantity by Faraday's law. Thus, it is independent of such factors as variations in ionization efficiency as a result, for instance, of column bleed.

Inter-laboratory/inter-method comparisons are a field in and of themselves. Well designed studies are highly expensive and have to take into account standards, preparative methods, sample splitting techniques and so on, as well as the instrumentation and parameters of instrument usage. Initial efforts are frequently discouraging. As an example the initial round of the multi center/method ESCOT study for 8 hydroxy 2'deoxyguanosine measurements initially returned values differing by a factor of 1000 with the higher values resulting from source artifacts in a gas chromatography-mass spectrometry (GC-MS) method.

Enzyme-linked immunosorbent assay techniques for this same analyte in urine are typically comparable to electrochemical methods for controls and standards but a factor of 2–5 higher in various disorders, whereas GC-MS techniques for this analyte in CSF have been reported as 10,000 times higher compared with electrochemical techniques.

### Statistical Analyses

The relationship between the purine analytes and clinical symptom ratings was examined for both time points. Descriptive statistics are provided in Yao et al. [8], [15] for the purine metabolites and tests of differences for these analytes between baseline and 4 weeks for patients, as well as comparisons between the analyte levels of age-matched healthy volunteers and patients at each time point. The focus of the present report concerns the relationships between ratings of clinical symptoms for this sample of FENNS patients and plasma levels of those metabolites that previously showed altered interactions within the purine pathway [8]: (1) single metabolites, including guanine, guanosine, xanthosine, xanthine, uric acid; (2) ratios of product to precursor, including guanine/guanosine, xanthine/guanine, uric acid/guanosine, uric acid/guanine, uric acid/xanthosine, xanthosine/guanosine, xanthosine/guanine.

Tests of the hypothesis that correlation coefficients involving the selected purine analytes and clinical symptoms were equal to zero were computed separately for the data recorded at baseline and the data measured at four weeks. Normality of data was estimated with Shapiro-Wilk Test of Normality (p>0.05); variables determined to approximate, or able to be transformed (ln, natural log) to approximate, normality were evaluated using parametric tests (paired-samples *t-*test; Pearson *r* correlation coefficient). Non-parametric tests (related-samples Wilcoxon Signed Rank Test; Kendall τ rank correlation coefficient) were used for those measures determined not normally distributed. Bonferroni correction was applied to control Type I error potentially associated with the multiple tests of our primary question, which involved 12 measures of purine catabolism at the two time points and improvement in patients' psychiatric disturbance (GAS, Global Assessment Scale) after one month of neuroleptic treatment: adjusted α for 24 comparisons: 0.05/24 = 0.0021; trend significance: 0.10/24 = 0.0042. Tests of the hypothesis that correlations at baseline and at 4 weeks were equal were additionally computed to screen for any baseline-to-four week between group (unmedicated versus medicated) correlation differences. This test for the difference between dependent correlations was computed using the procedure described by Bruning and Kintz [19].

## Results

**Figure 1:**
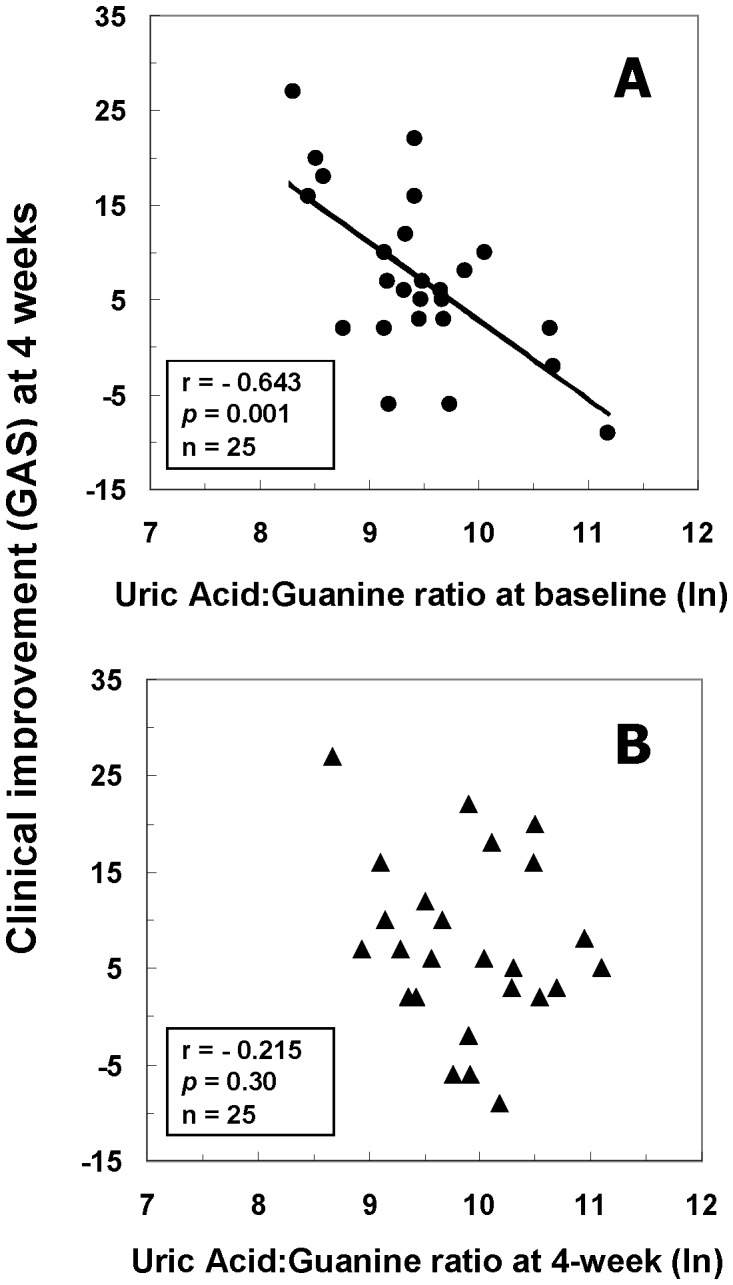
Associations between clinical improvement at 4 weeks and ratio of uric acid to guanine in first-episode neuroleptic-naïve patients with schizophrenia at baseline (A) or at 4-week (B) after antipsychotic treatment. Abbreviations: GAS, Global Assessment Scale; ln, natural logarithm.

The ratio of uric acid/guanine measured at baseline predicted improvement in clinical adjustment (GAS score at 4 weeks *minus* GAS score at baseline) after four weeks of treatment with neuroleptic drug. Following normalization of the data by natural log, this association was statistically significant (Pearson *r* = −0.643, *p* = 0.001), and involved an inverse relationship indicating that a lower initial proportion of product (uric acid) to precursor (guanine) was associated with *greater* improvement in clinical adjustment one month later (Figure 1A). In contrast, the association between the ratio of uric acid to guanine ratio measured at four weeks and clinical improvement (GAS scores) four weeks following initiation of neuroleptic treatment was not significant (Pearson *r* = −0.215, *p* = 0.30) (Figure 1B). The difference between these two correlations was statistically significant (*t* = −2.32, *df* = 22, *p*<0.05).

Correlation coefficients for the relationships obtained between the selected purine analytes and clinical symptoms are presented in Table 3 for the data recorded at baseline and in Table 4 for the data collected at four weeks.

**Table 3:** Correlation coefficients between purine metabolites and clinical assessments from first-episode neuroleptic-naive patients with schizophrenia at baseline before antipsychotic treatment.

Purine metabolites	GAS	BPRS-PSS	SANS	BPRS-TD	SI	MC	RM	SCMA
G*	0.414	−0.099	0.062	−0.132	−0.324	0.008	−0.205	−0.145
Gr†	0.151	−0.300	−0.014	−0.323	−0.298	0.035	−0.234	−0.203
Xant†	0.153	0.063	−0.118	0.069	−0.311	−0.025	0.092	0.203
Xan†	−0.014	−0.080	0.027	−0.038	−0.187	−0.025	−0.048	−0.123
UA*	0.033	−0.191	−0.121	−0.207	−0.402	−0.107	−0.317	−0.238
G/Gr†	0.224	0.136	0.108	0.127	0.102	−0.008	0.110	0.114
Xan/G*	−0.486	0.081	−0.175	0.119	0.145	−0.008	0.078	−0.045
UA/Gr*	−0.224	0.260	0.033	0.294	0.261	−0.107	0.278	0.274
UA/G*	**−0.643** ‡	−0.011	−0.217	0.039	0.050	−0.090	0.008	0.017
UA/Xant†	−0.170	−0.157	0.081	−0.181	0.102	−0.025	−0.206	−0.309
Xant/Gr*	0.172	0.055	−0.151	0.110	0.054	0.008	0.491	0.506
Xant/G†	−0.075	0.045	−0.141	0.099	−0.017	0.057	0.206	0.258

* Pearson correlation coefficient was calculated following transformation of the data (except MC) to natural logarithm.

† Kendall's tau rank correlation coefficient was measured when those data determined not normally distributed.

‡ Significance with *p* = 0.001 in boldface after the Bonferroni correction.

**Abbreviations**: G, guanine; Gr, guanosine; Xant, xanthosine; Xan, xanthine; UA, uric acid; GAS, Global Assessment Scale; BPRS, Brief Psychiatric Rating Scale; PSS, Positive Symptoms Scale; SANS, Scale for Assessment of Negative Symptoms; TD, Thought disorder; SI, Sensory Integration; MC, Motor Coordination; RM, Repetitive Motion; SCMA, Sequencing Complex Motor Acts.

**Table 4:** Correlation coefficients between purine metabolites and clinical assessments from first-episode neuroleptic-naive patients with schizophrenia 4 weeks after initiating antipsychotic treatment.

Purine metabolites	GAS	BPRS-PSS	SANS	BPRS-TD	SI	MC	RM	SCMA
G*	0.088	0.021	−0.215	0.090	−0.260	−0.108	−0.146	−0.072
Gr*	−0.067	−0.004	−0.178	0.086	−0.163	0.012	−0.088	−0.141
Xant†	0.456	−0.186	−0.222	0.011	−0.146	0.029	−0.064	−0.094
Xan†	−0.085	−0.111	−0.051	−0.166	−0.393	−0.264	−0.198	−0.127
UA*	0.061	−0.028	−0.065	−0.015	−0.343	−0.108	−0.070	0.006
G/Gr*	0.108	0.021	−0.099	0.008	−0.104	−0.147	−0.059	0.028
Xan/G†	−0.297	−0.071	0.330	−0.173	0.142	−0.029	0.057	0.072
UA/Gr†	0.163	0.081	0.108	−0.068	−0.096	−0.029	0.090	0.072
UA/G†	−0.215	0.038	0.303	−0.008	0.128	0.069	0.165	0.112
UA/Xant†	−0.312	0.143	0.070	0.015	−0.117	0.147	−0.002	0.028
Xant/Gr†	0.436	−0.098	0.029	−0.019	0.066	0.029	0.069	0.072
Xant/G†	0.175	−0.126	0.082	−0.098	0.192	−0.010	0.100	0.072

* Kendall's tau rank correlation coefficient was measured when those data determined not normally distributed.

† Pearson correlation coefficient was calculated following transformation of the data (except those data from BPRS-TD, MC, and SCMA) to natural logarithm.

**Abbreviations:** See footnote of Table 3.

**Figure 2:**
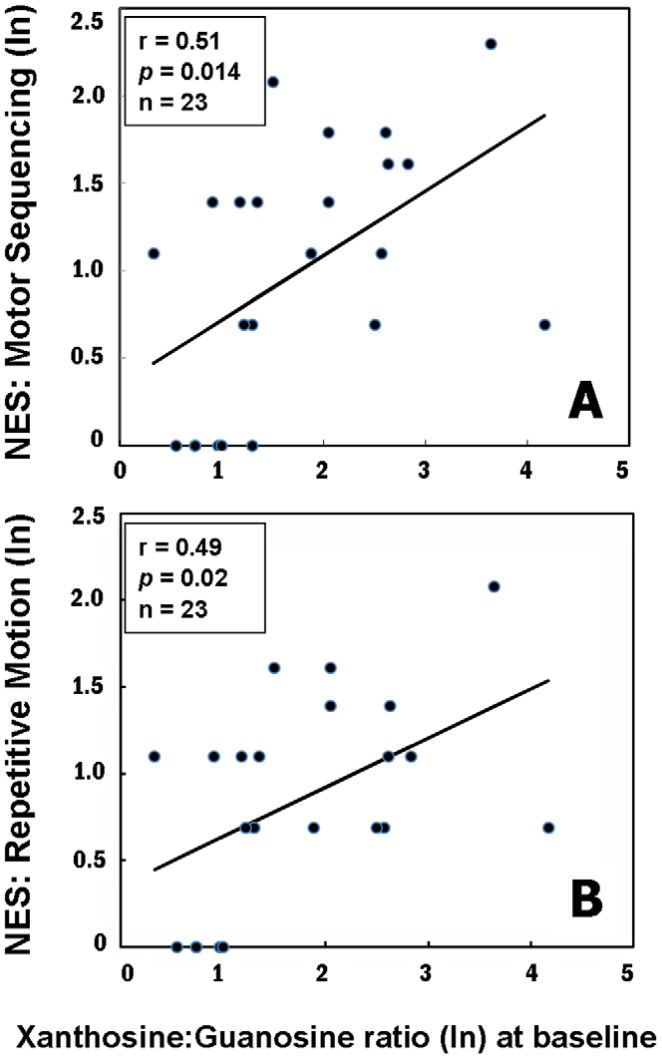
Pearson correlations between Neurological Evaluation Scale (NES) and ratio of xanthosine to granosine in first-episode neuroleptic-naïve patients with schizophrenia (FENN-SZ) at baseline. A, Motor sequencing; B, Repetitive motion. All data were transformed to natural logarithm (ln). The NES data were available at baseline from 23 FENN-SZ.

**Figure 3:**
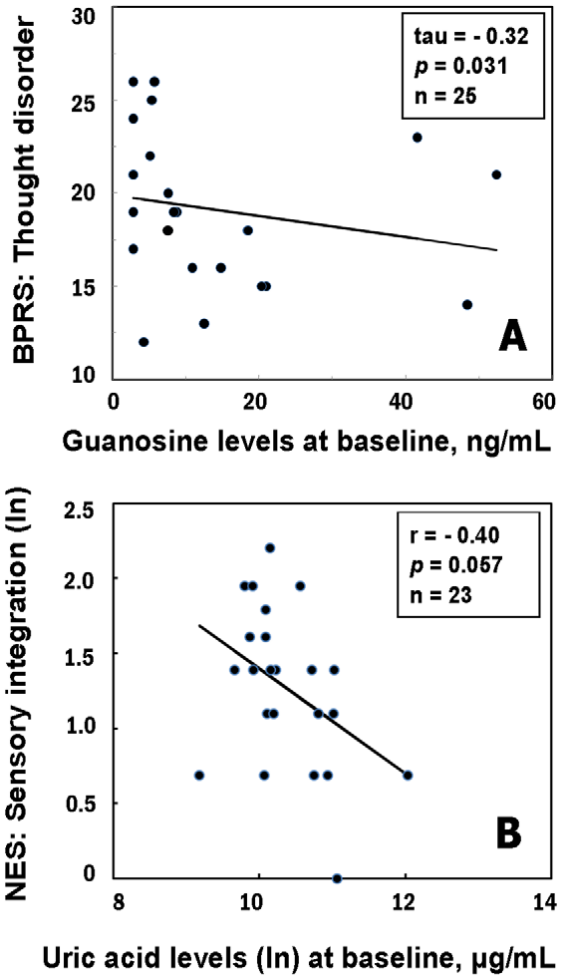
Kendall tau rank correlations between BPRS-thought disorder and guanosine levels (A), and Pearson correlations between NES-sensory integration and uric acid levels (B) in first-episode neuroleptic-naïve patients with schizophrenia (FENN-SZ) at baseline. The BPRS and NES data were available at baseline from 23 FENNS-SZ.

Findings from our exploration of associations among purine metabolites and specific symptom dimensions are additionally suggestive about domains of behavior that may be influenced by an imbalance in purine catabolism. Because of the exploratory nature of these latter analyses, *p*-levels were not corrected. Results showed that the ratio of xanthosine to guanosine at baseline was positively correlated with the neurological symptom involving the sequencing of complex motor actions (Figure 2A). This ratio (xanthosine∶guanosine) was also correlated with a factor that represents a composite of repetitive motor responses [20] (Figure 2B). Relationships were additionally observed between baseline guanosine levels and thought disturbance (Figure 3A), and between baseline uric acid levels and sensory integration (Figure 3B).

## Discussion

### Homeostatic imbalance of purine catabolism in schizophrenia

Increasing evidence suggests that mitochondrial pathology and oxidative stress may be one of the critical components in the pathophysiology and treatment outcome of schizophrenia [1]–[8]. Purine catabolism may contribute to mitochondrial antioxidant defense by producing the antioxidant uric acid [9]. Failure to maintain elevated xanthine and uric acid occurred contemporaneously with progressive mitochondrial dysfunction. On the other hand, increased levels of uric acid may be considered as a marker of oxidative stress [21], [22] due to accumulation of reactive oxygen species [23]. Therefore, uric acid can be served as both anti- and prooxidant in the AODS.

In man, uric acid is the final product of purine catabolism [24], which has been implicated as a risk factor and cause of numerous pathological conditions. Abnormally high plasma (or serum) uric acid has been related to cardiovascular disease, gout, hypertension, and renal disease, whereas low levels of plasma (or serum) uric acid have been linked to Alzheimer's disease, multiple sclerosis, optic neuritis, and Parkinson's disease [6]. Previously, we have also demonstrated that plasma levels of uric acid are reduced in chronic [25] as well as first-episode neuroleptic-naïve patients with schizophrenia [26]. Although some studies have indicated that uric acid may play a role in the development or progression of such diseases, it remains unclear whether an increased uric acid contributes to the cause or is simply a consequence of these pathologic conditions [27].

Moreover, in healthy control subjects, there exist tight product-precursor correlations within purine catabolism [8]. Interestingly, some of these correlations (i.e., conversion of hypoxanthine to xanthine) persist across disease or medication status, others (i.e., conversions of guanosine to xanthine, and xanthine to uric acid) are lost among these same FENNS patients [8]. Similar findings of lacking a control mechanism used by healthy control subjects were also demonstrated in the tryptophan pathway from these same patient group [15], indicating some metabolite interactions within purine catabolism were altered in FENNS patients. Taken collectively, purine catabolism appears to be a homeostatic response of mitochondria to oxidative stress and may play protect against progressive mitochondrial dysfunction in schizophrenia. Thus, our data as well as findings from other investigators are consistent with the notion of free radical-mediated neurotoxicity in schizophrenia [1]–[6].

### Purine metabolites and clinical improvement

Improvement in clinical functioning was associated with initial levels of uric acid and guanine in this sample of first-episode neuroleptic-naïve schizophrenia patients. The direction of this relationship indicates that a lower initial proportion of product (uric acid) to precursor (guanine) was associated with *greater* improvement in clinical functioning one month later. Thus, the lower the level of a patient's initial ratio of uric acid to guanine, the greater their overall clinical improvement at one month.

The initial severity of clinical dysfunction may be important to this relationship. As a group, the average level of clinical functioning reflected impairment at both time points, with mean values (<40) falling within the range typically observed for former inpatients likely to be readmitted to hospital [10]. Descriptively, degree of clinical improvement achieved by the patient group in the present study represented an increase from *“Unable to function in almost all areas …”* at baseline to *“Major impairment in several areas …”* one month later (GAS anchor points [10]). It may be appropriate, therefore, to qualify interpretation of findings based on this degree of severity.

### Purine metabolites and neurological symptoms

Neurological abnormalities are observed in schizophrenia patients even at the time of their first episode of psychosis and before the initiation of antipsychotic drug treatment [20], [28]–[31]. Moreover, neurological signs are correlated with clinical symptoms in unmedicated patients [32]. Significant heritability, or familial influence, has also been reported for several aspects of neurologic-related responding [33]. This overall pattern of findings has led some researchers to suggest that neurological abnormalities may represent a biological marker of schizophrenia risk. Finally and importantly, neurological signs are predictive of neuropsychological and cognitive performance in schizophrenia patients and healthy individuals [20], [34], and related to measures of neuroanatomical regions that are relevant for some of the cognitive deficits associated with this mental disorder [29], [31]. Findings from our present study suggest such interrelationships are also associated with alterations in purine catabolism in schizophrenia.

Purine metabolites were also linked to neurological and cognitive symptoms in our exploratory analyses based on this sample of FENNS patients. Firstly, motor responses recorded at baseline were associated with initial or baseline level of the ratio of xanthosine to guanosine. As shown in Figure 2A and 2B, the direction of these relationships indicates that a higher initial proportion of product (xanthosine) to precursor (guanosine) was associated with *greater* impairment in motor tasks. Thus, the higher a patient's initial or baseline ratio of xanthosine to guanosine, the greater his or her motor impairment was before initiating treatment with antipsychotic medications. Secondly, sensory processing was predicted by baseline level of uric acid. The direction of this relationship, seen in Figure 3, suggests that lower levels of uric acid were associated with *greater* impairment in sensory processing tasks.

A commonality among these neurological symptoms is the degree of complexity of response that is required by the assessment tasks. In one of the tests that tap the ability to sequence complex motor acts, for example, the individual is asked to use a steady rhythmic pattern to touch the table first with his/her fist, then with the edge of his/her hand, and then with the palm of his/her hand. The individual is told to repeat this sequence 15 times [13]. The sensory tasks also demand complex processing and responding by requiring the integration of auditory, visual, and tactile stimulation followed by an action that involves either a verbal or other motor response, such as the placement of a hand or pointing of finger. Our group has previously documented highly statistically significant associations between these neurologically-based behaviors and standard neuropsychological tests of memory and intelligence in patients with first-episode psychosis who were neuroleptic-naïve at the time of testing [20]. The present report, which also involved FENNS patients, provides evidence of linkages between those same neurologically-based behaviors and metabolites within the purine pathway, which is closely linked to a series of enzymatic and non-enzymatic components collectively referred to as the AODS.

Associations among uric acid, which represents an endpoint of purine metabolism in humans, and intelligence and achievement were first reported in the modern research literature over five decades ago [35]–[37]. Of interest is the mention by Barrera et al. [38] of a connection made between uric acid and intelligence that appeared in the published literature as early as the 1600s. Present day discussions include the hypothesis that the relative increase in levels of uric acid in humans, compared to other mammals, may have conferred an evolutionary advantage [39]. Although attention to this topic declined during the mid-1980s and throughout much of the 1990s, recent reports are suggestive of a growing interest in the contribution of alterations in purine metabolism to illness and disability. Related evidence includes associations reported between low levels of uric acid and risk for Parkinson's Disease (PD) [40], and between low levels of uric acid and reduced performance on neuropsychological tests of intelligence and attention in patients with this disease [41]. By contrast, associations have been reported between elevated levels of uric acid and motor behaviors, including increased uric acid and hyperactive behaviors in normal preschool children [38], increased uric acid and increased locomotor activity in rats [42], and an abnormal increase in level of uric acid and psychomotor excitation in schizophrenia patients during acute illness [43]. Beyond mammals, other evidence implicates a role for purines, including hypoxanthine and uric acid, in the sensitivity to external stimuli and motor responses of insects [44]. In combination with these findings from the research literature, our data are suggestive of some optimal level of purine byproducts and dynamics in the integrity of intelligence and manifest behavior.

### Limitations

The present study is mainly involved in the purine metabolites of peripheral blood samples. Whether such an association between optimal levels of purine byproducts and dynamics in clinical symptoms is present in the brain awaits further investigation.
